# Accelerating Virtual Health Implementation Following the COVID-19 Pandemic: Questionnaire Study

**DOI:** 10.2196/32819

**Published:** 2022-05-16

**Authors:** Melissa Stahl, James Cheung, Kevin Post, James P Valin, Ira Jacobs

**Affiliations:** 1 The Health Management Academy Arlington, VA United States; 2 Avera Medical Group Sioux Falls, SD United States; 3 The Sisters of Charity of Leavenworth Health System Inc Broomfield, CO United States; 4 Pfizer Inc New York, NY United States

**Keywords:** virtual health, eHealth, mHealth, telemedicine, telehealth, COVID-19, health system, care delivery, strategy, business model

## Abstract

**Background:**

The COVID-19 pandemic accelerated drivers for virtual health adoption and triggered the US federal government to implement regulatory changes to reduce barriers to virtual health implementation. Consequently, virtual health solutions have been increasingly adopted, and health systems in the United States have been reorganizing their care delivery process with unprecedented speed.

**Objective:**

This study aimed to assess and make recommendations on the strategy, business model, implementation, and future considerations for scaling and sustaining virtual health solutions based on the views of executives from the largest health systems in the United States.

**Methods:**

In September 2020 and October 2020, the Health Management Academy conducted 29 quantitative surveys and 23 qualitative interviews involving 58 executives from 41 of the largest health systems in the United States. Participating health systems were approximately equally distributed across size categories (small, medium, and large, defined as annual total operating revenue US $2-3 billion, $3-6 billion, and >$6 billion, respectively) and US Census Bureau regions (Northeast, Midwest, South, and West).

**Results:**

Based on the Health Management Academy’s assessment of approaches to governance, financing, data infrastructure, and clinical integration of virtual health, most participating health systems (13/24, 54%) had a mid-stage level of maturity in virtual health implementation. Executives reported the pandemic is forcing health systems to re-examine strategic priorities; the most commonly raised key impacts were increased access (15/21, 71%) and flexibility (10/21, 48%) as well as lower costs of care delivery (9/21, 43%). Most executives (16/28, 57%) reported their organization had a defined budget for virtual health, and many noted that virtual health is best supported through value-based payment models. Irrespective of health system maturity, reimbursement was consistently rated as a key challenge to virtual health scaling, along with patient access to and understanding of virtual health technology. The success of virtual health implementation was most commonly measured by patient satisfaction, health care provider engagement, and proportion of health care providers using virtual health solutions (reported by 7/8, 88%; 6/8, 75%; and 7/8, 75% of information technology executives, respectively). Almost all health systems (27/29, 93%) expect to continue growing their virtual health offerings for the foreseeable future, with user-friendliness and ease of integration into the electronic medical record as key factors in making go-forward decisions on virtual health solutions (each selected by 9/10, 90% executives).

**Conclusions:**

The increased demand for virtual health solutions during the COVID-19 pandemic is expected to continue postpandemic. Consequently, health systems are re-evaluating their current platforms, processes, and strategy to develop a sustainable, long-term approach to virtual health. To ensure future success, health system leaders need to proactively build on their virtual health solutions; advocate for payment, site flexibility, and reimbursement parity for virtual health; and demonstrate continued engagement and boldness to evolve care beyond established models.

## Introduction

Virtual health is a rapidly evolving field, encompassing a range of information and communication technologies and forms of care delivery. Although there is no static and universally agreed definition of virtual health, the World Health Organization has defined eHealth in the following way: “the cost-effective and secure use of information and communications technologies in support of health and health-related fields, including health-care services, health surveillance, health literature, and health education, knowledge and research” [1].

Virtual health, as well as telemedicine or telehealth, could be considered as components of eHealth, with virtual health focused on modalities of care delivery. For the purposes of this research, we defined virtual health to include the following components.

- Live (synchronous) videoconferencing: a 2-way audiovisual link between a patient and health care provider
- Store-and-forward (asynchronous) care delivery: transmission of a recorded health history to a health care provider or patient
- Remote patient monitoring: the use of connected electronic tools to record personal health and medical data in one location for review by a health care provider in another location, usually at a different time
- Mobile health (mHealth): health care and public health information provided through mobile devices. The information may include general educational information, targeted texts, and notifications about disease outbreaks.

Over recent years, there have been trends toward an increase in virtual care solutions for patients [2]. However, prior to the COVID-19 pandemic, health systems across the United States faced several barriers to the expanded use of virtual health, including a lack of reimbursement, uncertainty around workflow integration, resistance to change, and concerns about quality and efficacy [3,4]. The COVID-19 pandemic accelerated drivers for virtual health adoption, as maintaining continuity of care during “stay at home” orders necessitated increased use of virtual health to minimize exposure to and spread of the virus during care delivery. The pandemic also triggered the US federal government to implement multiple regulatory changes to reduce barriers to virtual health implementation. Pandemic emergency orders allowed for cross-jurisdictional recognition of licensure, enabling health care providers licensed in one state to work in any other state [5-8]. The Coronavirus Preparedness and Response Supplemental Appropriations Act of 2020 waived many of the geographic and site restrictions on Medicare reimbursement [9]. The US Department of Health and Human Services loosened enforcement of some rules under the Health Insurance Portability and Accountability Act of 1996 during COVID-19, giving health care providers more flexibility in the use of telehealth solutions [10]. Consequently, virtual health solutions have been increasingly adopted, and health systems in the United States have had to reorganize their entire care delivery process with unprecedented speed [11,12].

Although the pandemic has catalyzed significantly higher virtual health utilization, health care providers and academics in the field are predicting a fundamental, enduring shift in how health systems deliver care [12-14]. Although health systems scaled virtual health out of necessity, innovating to meet the needs of an immediate crisis is different from sustainable, long-term transformation. Consequently, health systems are beginning to re-evaluate how the virtual health strategies implemented during the COVID-19 surge in virtual health utilization will meet the needs of the system, health care providers, and patients going forward. The pandemic quickly demanded that patients, health care providers, and health systems adapt virtual care at an unprecedented pace of change. Continuing to manage this rate of change will continue to be a challenge postpandemic. Maintaining these elevated levels of virtual health adoption among health systems into the future will depend on key factors, including sustained payment mechanisms, regulatory flexibility around site-of-service requirements, and development of a virtual care delivery model that wins the confidence of patients, health care providers, and health systems with regard to quality, safety, and cost. With 637 separate health systems across the United States [15], sharing learnings and best practices between health care providers is important for ensuring consistency and equity in health care delivery across the nation. Sharing information can also assist health systems with decision-making and implementation of the most effective solutions. Some of these learnings may also be applicable to health care systems in other countries.

In this study, we aimed to assess and make recommendations on the strategy, business model, implementation, and future considerations for scaling and sustaining virtual health solutions based on the views of executives from the largest health systems in the United States.

## Methods

### Overview

The Health Management Academy (referred to herein as The Academy) comprises over 3500 members, including C-suite executives and principal leaders from more than 150 of the largest health systems in the United States (each with an annual operating revenue of at least US $2 billion), as of July 2021 [16]. In September and October of 2020, The Academy distributed a survey and interview request to members with clinical, operational, or informatics roles with role titles that included Chief Medical Officer (CMO), Chief Nursing Officer, Medical Group President, Chief Operating Officer, Chief Financial Officer, Chief Information Officer, Chief Medical Information Officer, and Chief Nursing Information Officer.

The survey and interviews included questions on health systems’ strategic approach, implementation, and future outlook on their virtual health modalities. Surveys were distributed by email and included 18 multiple choice questions divided into 4 sections: general quantitative survey, clinical, strategy and operations, and information technology (IT)/data (Multimedia Appendix 1). The questions included under the General Quantitative Survey section were given to all participating executives, whereas the sections on clinical, strategy and operations, and IT/data were sent to targeted executives with expertise on those topics. Interviews ran for 30 minutes to 60 minutes, were conducted virtually through Microsoft Teams, and included questions based on a qualitative interview guide containing 31 questions divided into 6 sections: strategy, structure, implementation, finance, data, and consumerism (Multimedia Appendix 2). Questions from each section were asked as relevant to the interviewee’s role; for example, only C-suite executives in IT were asked questions in the data section. Executives from the same health system were allowed to attend the same interview if preferred.

The Academy collected responses and analyzed results. For the quantitative survey data, percentages were calculated for each question, with the total number of responses to each question as the denominator. For the sections on clinical, strategy and operations, and IT/data, the denominator was the number of executives with the relevant experience who answered each question. If a respondent skipped a question, then the denominator for that question would be reduced by 1. Due to the small sample sizes involved, statistical analyses of survey data were not feasible. Responses from the interviews were qualitatively assessed, and observations were summarized in the report. Quotes were blinded and included as spoken by interview participants.

**Figure 1:**
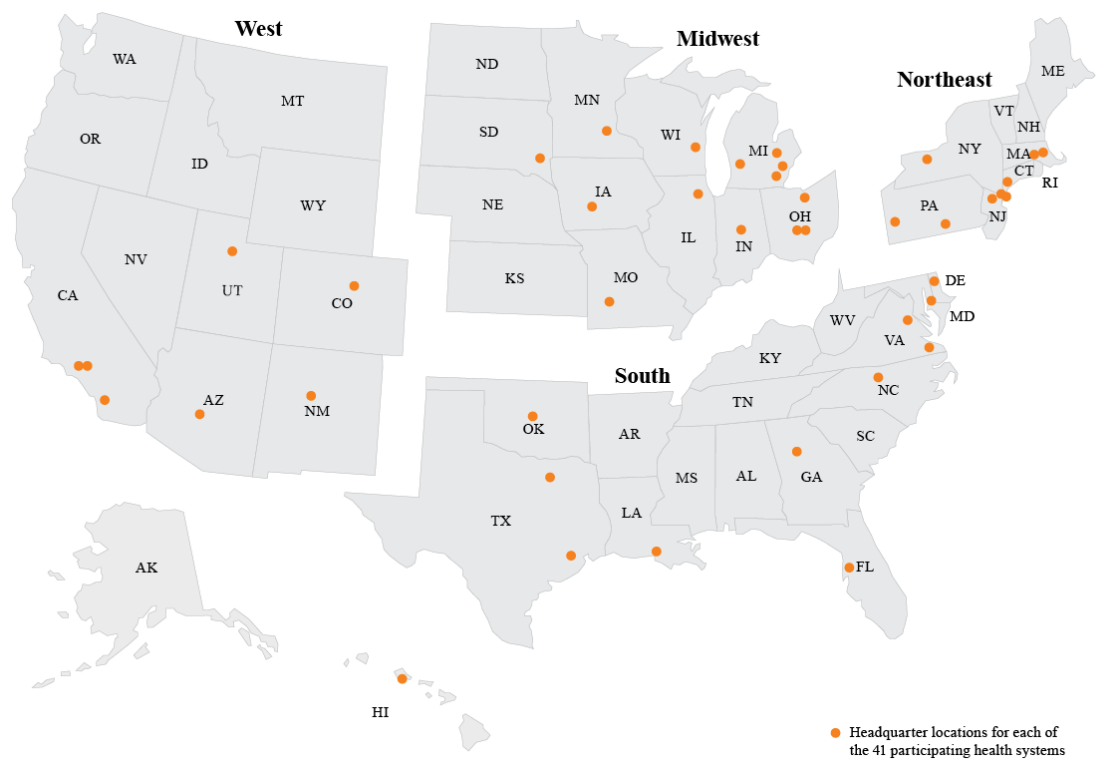
Geographic regions and headquarter locations of participating health systems; includes one health system with 2 headquarter locations identified.

**Figure 2:**
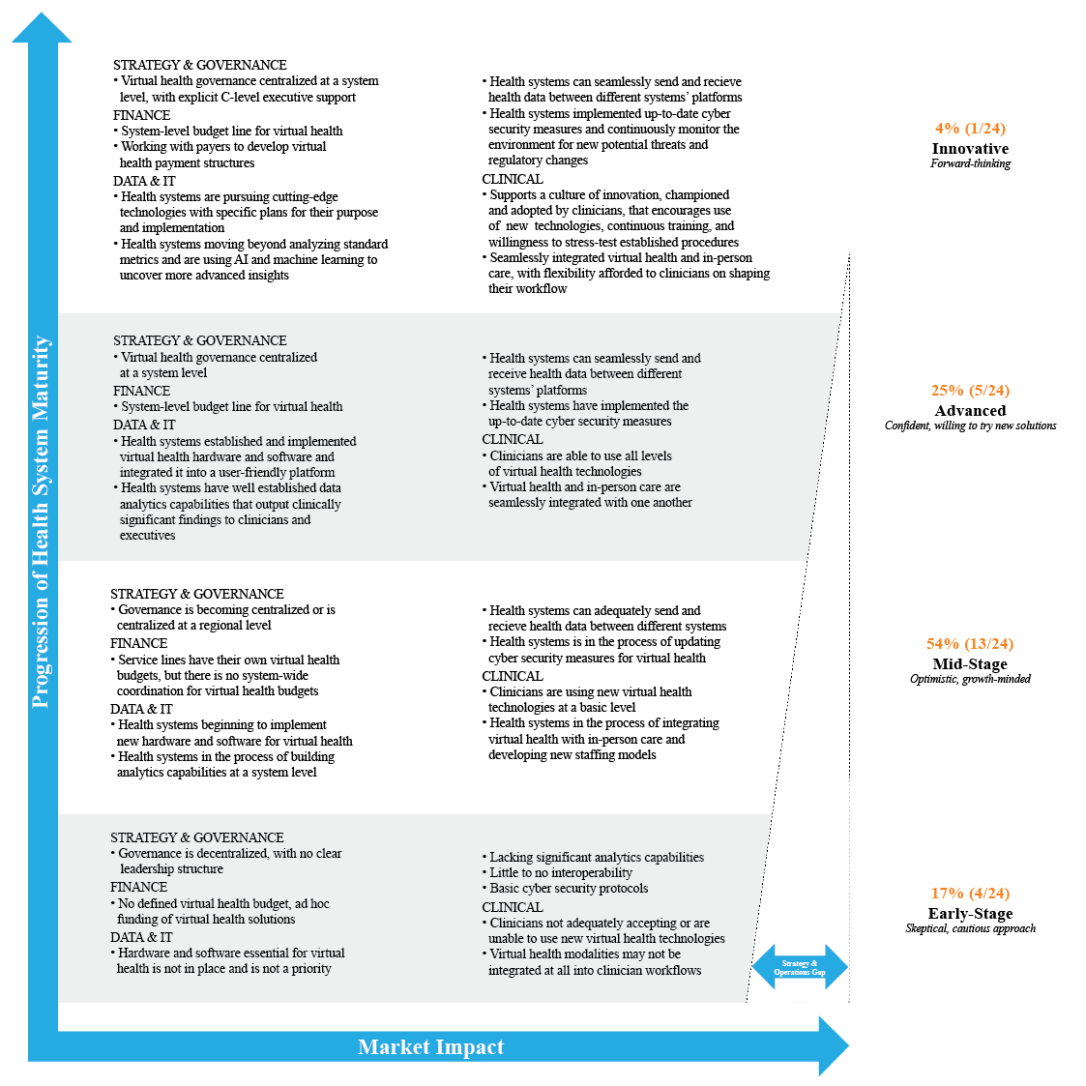
Overall health system maturity across virtual health programs. AI: artificial intelligence; IT: information technology.

Data were segmented by health system size, geographic region, percent of revenue from government pay, and virtual health maturity. Health system size was defined based on annual total operating revenue (TOR), including all revenue from both patient care and health plan if applicable; large systems were those with >US $6 billion TOR, medium systems were those with $3-$6 billion TOR, and small systems were those with <$3 billion TOR. Geographic regions were based on the US Census Bureau regions [17] and were defined as Northeast, Midwest, South, and West (Figure 1). Government pay was defined as the proportion of payer mix coming from Medicare, Medicaid, or other government sources, with high and low categories of greater than or less than the median of the survey group, respectively. Virtual health maturity was defined and assessed by The Academy based on responses to survey and interview questions in 4 categories: virtual health governance, finance, data/IT, and clinical. General features of health systems at early stage, mid-stage, advanced, and innovative levels of virtual health maturity are shown in Figure 2. Selected subgroup analyses are presented for qualitative comparison.

### Ethical Considerations

This research was considered exempt from institutional research board review according to the US Department of Health and Human Services, Office for Human Research Protections, §46.101, (b)(2) [18] because it involved survey procedures, and the information obtained could not be linked to the participants and did not place them at risk.

## Results

### Participating Health Systems

Study participants included 58 executives from 41 separate health systems in the United States. There were 29 quantitative survey responses and 23 qualitative interviews (some interviews involved more than 1 executive).

**Figure 3:**
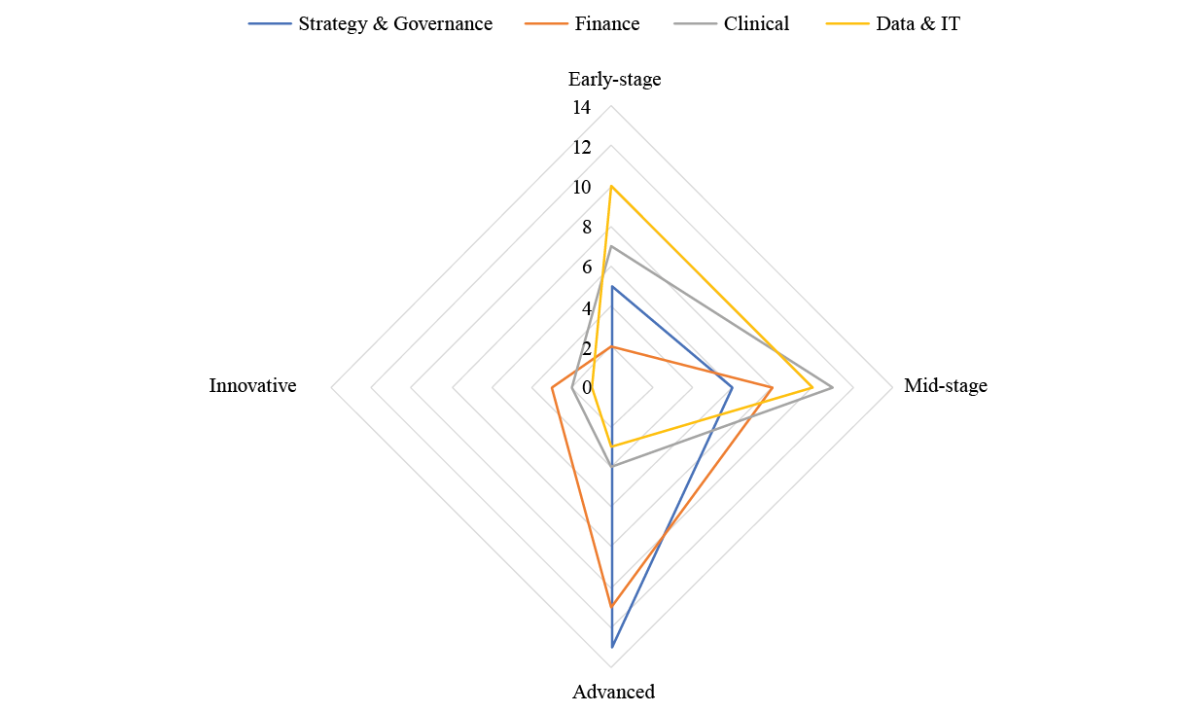
Virtual health maturity by domain. IT: information technology.

Participating health systems represent a mean TOR of US $5.5 billion. Participating health systems were approximately evenly distributed across the size categories: 32% (13/41) were large, 39% (16/41) were medium, and 29% (12/41) were small. Participating health systems were also approximately evenly distributed across the different US regions: Northeast (9/41, 22%), Midwest (13/41, 32%), South (11/41, 27%), and West (8/41, 20%). The median government pay across these systems was 42% (range 23%-66%). Based on their approaches to governance, financing, data infrastructure, and clinical integration of virtual health, most participating health systems (13/24, 54%) were categorized as being at a mid-stage level of overall maturity in virtual health implementation (Figure 2). Health systems tended to be at a more advanced level of maturity in their approach to strategy/governance and finance and less mature in their data and IT infrastructure and clinical integration (Figure 3).

### Virtual Health Strategy

Prior to the COVID-19 pandemic, most health systems had implemented some form of virtual health solution for specific use cases, most commonly tele-stroke, tele-intensive care unit, or within behavioral health. However, most health systems did not have defined infrastructure, processes, or strategies to leverage virtual health on a large scale. Most health systems reported a neutral to conservative approach to pursuing virtual health modalities pre-COVID-19 (60% to ≥70% across modalities). Reimbursement difficulty and lack of integration with the electronic medical record (EMR) were cited as the main barriers to virtual health implementation.

Executives reported that the pandemic is forcing health systems to re-examine strategic priorities, and executives raised multiple key impacts on health systems and health care providers. In light of the virtual health “new normal,” health systems are forced to re-examine the cost structure of their business model and the composition and skills of their workforce, as well as the need for brick-and-mortar facilities. There is also a push for health systems to incorporate insights from consumer-facing industries (eg, banking, finance) in digital front door strategies. Health systems and health care providers are being pushed to develop a strategy to partner with patients to design and implement virtual health solutions from a consumer lens, address the social determinants of health that impact patient access to virtual health, and ensure equity and access in moving beyond traditional (eg, phone, video) telehealth services. Health care providers are also being forced to adopt virtual health and learn how to modify their workflows accordingly.

In order to scale existing virtual health modalities to meet surging demand, many health systems found building on their existing platforms—regardless of how small—was the best choice for rapidly scaling virtual health to meet the needs of their patients and health care providers:

We have had a telehealth option since 2012, and the growth had historically doubled each year from a very small base. But that growth is now exponential—we went from about 70,000 virtual health visits a year in 2019, to nearly 70,000 per week by early April (2020).

Chief of Medical Technology

Health system executives reported that, during the COVID-19 pandemic, immediate priorities included maintaining patient and staff safety by keeping health care providers and patients remote whenever possible and sustaining revenues to support the business. As health systems look to develop a long-term strategy for virtual health, executives are beginning to set new goals for enterprise-level virtual health and, in particular, increasing flexible, consumer-friendly care; improving access and expanding market share; broadening the suite of digital health technologies; and building new virtual health business models.

**Figure 4:**
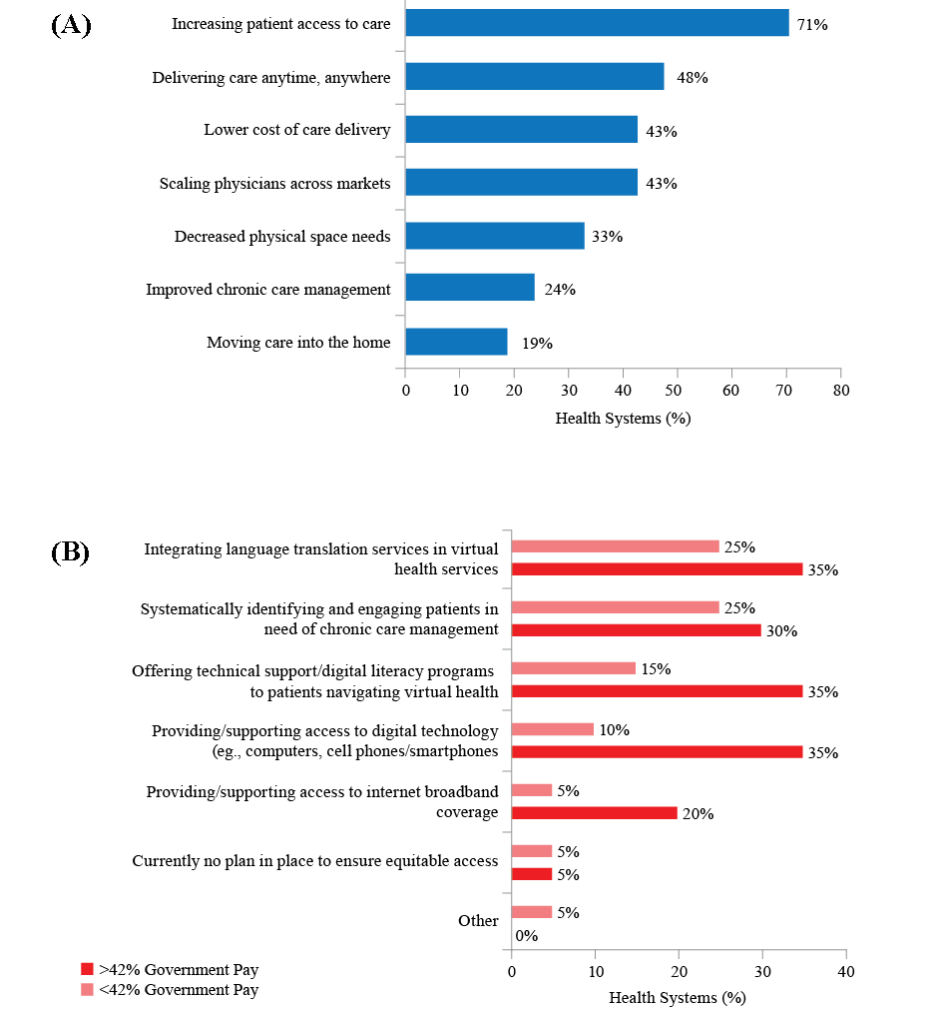
Impacts and strategies for virtual health implementation, including (A) areas in which participating health systems expect the greatest impact from virtual health (n=21) and (B) strategies in place to support equitable access to virtual health (n=20); the segmenting point of 42% government pay represents the median across participating health systems.

Areas in which executives expect virtual care to have the greatest impact include increasing access and flexibility of care delivery and lowering costs (Figure 4A). It will be essential for health systems to leverage technology to develop a model of a continual ongoing relationship with the patient across the health care journey of their lifetime, as compared with a previous model of episodic care. As virtual health expands, executives are prioritizing equity in access and outcomes. Health systems do not want virtual health to exacerbate existing divides in patients’ ability to access and benefit from care and are taking steps to monitor and address equity concerns (Figure 4B).

Many health system executives considered virtual health to form part of both their care delivery strategy and consumer strategy. This is because improved access, flexibility, and convenience are increasingly sought by consumers, and virtual solutions may assist health systems in meeting these expectations. However, health system executives recognize the opportunity to improve the consumer-friendliness of their current virtual health systems. Most executives from large health systems (5/9, 55%) rated their organization’s virtual health systems as either consumer-friendly or very consumer-friendly. For medium and small health systems, only 20% (2/10) and 14% (1/9) of executives, respectively, rated their virtual health systems as consumer-friendly or very consumer-friendly.

### Virtual Health Business Model

**Figure 5:**
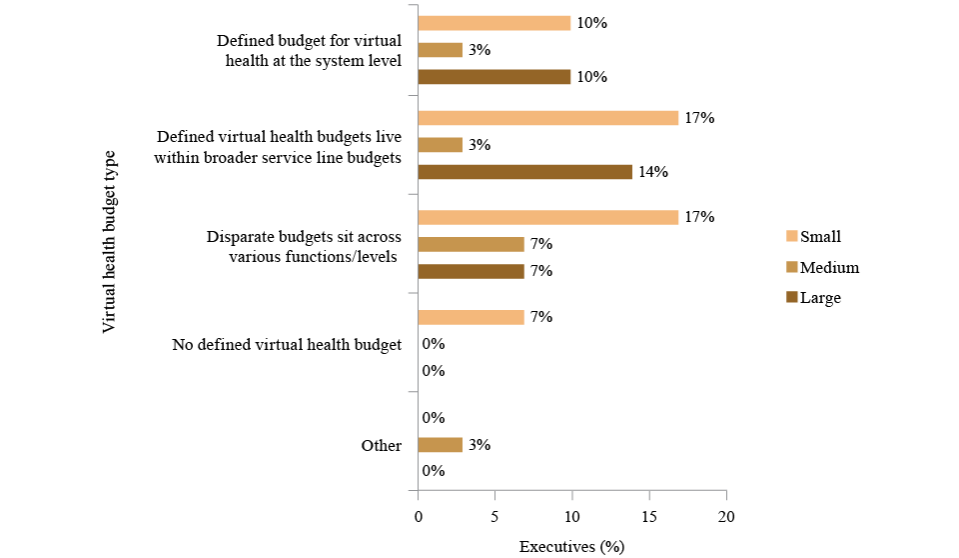
Virtual health budget types by health system size, as provided by executives (n=28).

Health systems will likely focus on development of a hybrid model of virtual and in-person care, with efforts focused on population health and value-based reimbursement. Most executives (16/28, 57%) reported their organization had a defined budget for virtual health (Figure 5).

Many executives expressed concern that a retraction to a prepandemic lack of payment and site-of-care restrictions on virtual health would significantly detract from their ability to deliver care virtually:

If payment parity for virtual visits goes away, expenses will have to decrease to meet the corresponding decrease in that revenue stream... We’re not going to stop doing virtual, because we believe it’s the right thing to do for our patients, and it’s about continuing to evolve with the future of healthcare.

Senior Vice President, Finance

Executives saw virtual health as a strategy best supported through a value-based payment model:

Going forward, we’re trying to get to a more value-based world where we’re only bringing patients onsite if they need complex care. That’s a large transformation that is underway, and virtual is an important component of that, but there’s still a long way to go.

Chief Medical Information Officer

Most health systems in the United States are predominantly fee-for-service and have only a small proportion (an average of 29%) of revenue coming from value-based payment models. Executives reported that their organization would not shift to a fully value-based model until a larger proportion of revenue comes from these models.

The [value-based payment] tipping point is probably 40%, but we’re not near that now; we’re at 10% or less, and many value-based arrangements start with fee for service.

Senior Vice President and CMO of Ambulatory Service

Health systems are restructuring their operations to adapt to the increased use of virtual health. For example, most health systems (6/10, 60%) intend to use technology to automate certain tasks associated with virtual services, such as clinical documentation during virtual visits. This increased automation can also assist with cost management. Other strategies to address cost management in response to the shifting business model include reducing or consolidating the physical clinic’s square footage (5/10, 50% of health systems), adjusting the workforce by reducing or reassigning staff (3/10, 30%), and redesigning health care provider compensation (2/10, 20%). Health systems may also be able to manage costs by aligning and partnering with other health systems to provide financial support for necessary technologies.

Health system executives stressed that the federal government’s actions to maintain payment for virtual health and promote flexibility in care delivery during the pandemic will need to be sustained for health systems to continue to provide virtual care. These responders were optimistic about these benefits being extended, and some health systems have developed “legislative teams” willing to align efforts with other health systems to help drive government support, at both state and federal levels, for sustained reimbursement for virtual care. Despite these efforts, uncertainty remains:

The maintenance of virtual visits is dependent on a payment model remaining in place for them, I can’t stress that enough. If there isn’t payment parity between virtual and in-person, we’ll have to re-examine how to manage our costs.

President, Medical Group

### Implementing Virtual Health Solutions and Workflows

Health systems experienced a rapid surge in virtual health utilization during the peak of the COVID-19 pandemic; during spring and summer 2020, 58% of total visits to participating health systems were virtual. As in-person care resumed, the proportion of virtual visits declined, reaching 21% at the time of the survey (September-October 2020).

As they exit the COVID-19 surge, health system executives report a shift in focus to medium- and longer-term virtual health strategy, with key concerns of payment mechanisms and the ability to practice across state lines. Going forward, 67% (6/9) of participating health systems have a defined target for the percentage of health visits occurring virtually. Almost one-fourth (2/9, 22%) of health systems reported that they want 21% to 30% of all care to be delivered virtually going forward, and one-third (3/9, 34%) of health systems expect 11% to 20% of care delivery to be virtual.

**Figure 6:**
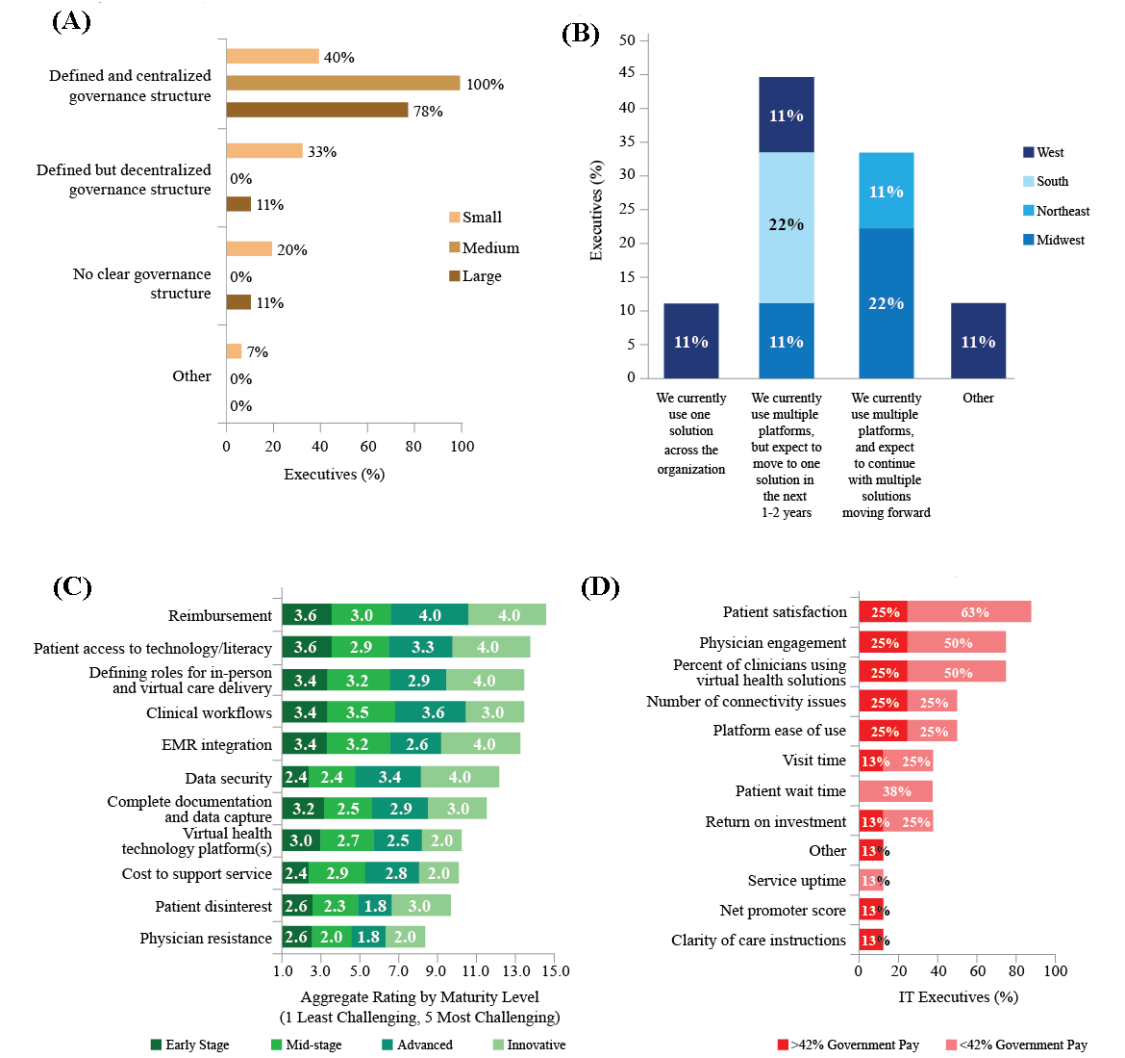
Features of virtual health implementation, as reported by executives, including (A) virtual health governance structure by health system size (n=29), (B) use of multiple virtual health platforms by region (n=8), (C) challenges to virtual health scaling by health system maturity level (n=29), and (D) metrics to measure the success of virtual health implementation, by proportion of government pay (n=8). EMR: electronic medical record; IT: information technology.

As health system executives consider their long-term virtual health strategy, many expect to leverage the governance structures established during the pandemic moving forward. For medium and large health systems, almost all executives reported that their organization had a centralized governance structure for virtual health implementation (Figure 6A). These central committees were typically composed of multiple key stakeholders and representative groups (eg, clinical leadership, regulatory, patient experience), and many executives reported their organization had a formal leadership role established for virtual health (eg, CMO of virtual health). Virtual health governance structures most commonly fell within the IT and/or population health division (14/29, 49% and 10/29, 34%, respectively), and most participating health systems created a dedicated virtual health committee or taskforce, which will remain post-COVID-19.

Key considerations around creating these new governance structures included centralization versus decentralization of authority, defined versus undefined accountability, and outsourcing versus insourcing committee members. High-maturity approaches tended to feature a multistakeholder governance structure that clearly delineated responsibilities for virtual health strategy and implementation.

During the COVID-19 surge in virtual health utilization, the higher a health system was on the virtual health maturity curve, the more likely they were to scale up existing solutions rather than implement new technology. Patient and health care provider usability was reported as being the primary criteria for selecting virtual health solutions. Health care provider acceptance and adoption of virtual health increased during the pandemic; however, health system executives reported less success in developing and integrating virtual health into existing clinical workflows in a sustainable and comprehensive way.

Health system executives and leaders are considering whether their existing EMR and IT capability will be positioned to sufficiently support and adapt to telehealth efforts moving forward or whether a partnership with an outside vendor would be a more viable option long term. Most (7/9, 77%) IT executives reported their organization currently uses multiple virtual health platforms; however, 44% (4/9) aim to streamline operations and move to one base platform in the next 1 year to 2 years (Figure 6B). Most health systems (19/29, 66%) used Epic as their primary virtual health software solution, and most (15/29, 52%) used Zoom as their primary synchronous video platform. All IT executives reported that their organization had at least some success in integrating virtual health data into the EMR, with 22% (2/9) rating this integration as very successful. There will need to be a focus on developing platforms that are friendly and affordable to patients and health care providers.

The expansion of virtual health has caused some organizations to consider reshaping their clinical pathways from being EMR-centric to more consumer-focused:

Our EMR plays an important role, but when we launched our virtual health app, we made the decision to shift away from EMR-focused processes to consumer-focused ones. What’s easy and intuitive for the consumer is not the same as the way the EMR wants you to do things.

Chief Strategy Officer

Executives were generally less confident in their IT capabilities (eg, interoperability, technology, data analytics) than some of the process components of virtual health implementation (eg, adoption by health care providers). Executives were most confident in their cybersecurity proficiency and the distinct virtual health technology solutions, with leaders rating their proficiency within these components as “at goal and advancing.” Executives noted that, to effectively implement virtual health enterprise-wide, it is critical to ensure that virtual health solutions effectively support both patients and health care providers with frictionless, accessible care at each step of the patient journey, from finding care to managing care, and paying for care:

Our digital front door aims to help patients solve their inconvenient pain points – where am I in my deductible? How much do I owe? To do that, we’re focused on empowering the patients to answer three questions: how do I find the care I need, how do I manage the care I need, and how do I pay for it.

Chief Strategy Officer

Some health system executives reported using virtual health to supplement and extend the reach of their existing care infrastructure. For example, one health system used physicians’ offices as specialty access hubs where the primary care physician could connect virtually with specialists in a single appointment. In another example, the health system’s virtual health platform was incorporated into ambulances, enabling emergency department staff to connect virtually and triage patients to the most appropriate care venue according to the level of care needed. Approximately 50% of the time, health care providers were able to make decisions to monitor patients at home, reducing the number of emergency department visits, and thereby reducing potential exposure to COVID-19 and conserving personal protective equipment.

Irrespective of health system maturity level, reimbursement was consistently rated as a key challenge to virtual health scaling, along with patient access to and understanding of virtual health technology (Figure 6C). To improve patient and health care provider understanding, health systems may need to develop and provide training for virtual care to both their patients and health care providers.

### Defining Metrics to Measure the Success of Virtual Health Implementation

Patient and health care provider satisfaction and health care provider engagement are the most commonly prioritized virtual health metrics among participating IT executives (Figure 6D). Health system executives reported a need to increase the tracking and measurement of virtual health–specific metrics.

Health metrics differed slightly between health systems with high and low government pay, with low government pay systems more likely to prioritize patient satisfaction and wait time (Figure 6D). Organizations used a variety of different metrics, including measures of patient and health care provider satisfaction, clinical and screening metrics, platform and follow-up metrics, and financial metrics. Health system executives commonly reported they have not yet settled on their key metrics but recognize their importance for virtual health validation.

Several health systems are using pilot projects to better evaluate the success of their virtual health initiatives on metrics such as total cost of care, readmissions, and follow-up rates. Health systems plan to build on these projects to understand how they can continue to optimize their use of virtual health. These pilot projects enable health systems to maintain the “fail fast” mentality that they developed during the pandemic.

Health system executives stated they have a narrow window of time to collect sufficient data on virtual health’s efficacy and efficiency compared with in-person care, in order to make the case to regulators and payers for why supporting virtual health’s financial sustainability is a must. There were some differences of opinion between executives on whether in-person measures can or should be transferred over to digital modalities.

### Future Considerations

Almost all health systems (27/29, 93%) expect to continue to grow most or all of their virtual health offerings for the foreseeable future:

What’s here to stay is the adoption of it, acceptance that care is not always in person. People have trepidation around payment, but what I’ve said is we're not going to have a choice. It's not going to matter. Health systems and physicians will learn how to adopt this, or they will go out of business or be purchased. You have maximum two to three years. If you're not aggressively adopting these services, you will fail.

Executive Director, Virtual Care

**Figure 7:**
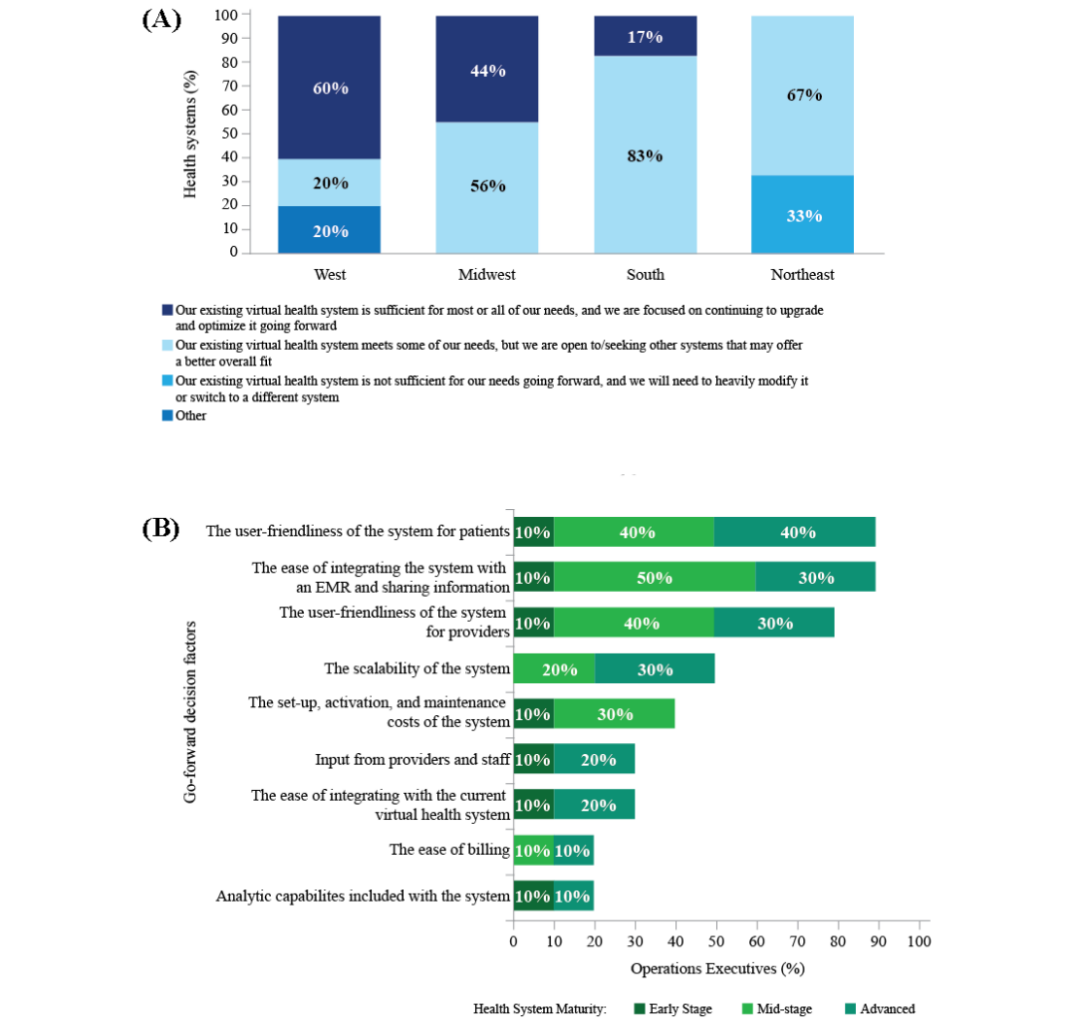
Future considerations for virtual health implementation, as reported by executives, regarding the (A) sufficiency of current virtual health systems by region (West: n=5; Midwest: n=13; South: n=11; Northeast: n=9) and (B) factors in making go-forward decisions on virtual health initiatives (n=10). EMR: electronic medical record.

Health system executives’ degree of satisfaction with their current virtual health system varied between US regions, with executives from health systems in the Western region most likely to rate the organization’s virtual health system as sufficient for most or all of their needs (Figure 7A). Many health systems are reevaluating existing virtual health infrastructure, with user-friendliness and ease of information sharing or integration with the EMR as the 2 most commonly reported key factors (9/10, 90% of operations executives) in making go-forward decisions on virtual health initiatives (Figure 7B). Other factors in making go-forward decisions included to ensure data-driven decision-making; maintain patient confidence in a trusting, confidential patient-health care provider relationship; and a focus on how to best leverage technology to support a hybrid model of virtual and in-person care. This will require both patients and health care providers to rethink their relationship and the journey of providing consumer-focused health care. Alternate payment models driven by population health and value-based reimbursement will require adaptability and change in health care provider contracts.

Executives raised a number of key challenges moving forward, including transparency and flexibility in health care provider licensing, maintaining the “move fast” cultural shift, integrating patient-generated health data with the EMR, determining which types of visits should be virtual versus in-person, and maintaining relationships between health care providers and their patients. Other unknowns and challenges included the pandemic trajectory, aid and politics, macroeconomic factors, demand for services, impact of regulations, and operational resilience. Addressing burnout and health needs of health care providers were also identified as issues going forward, with most (13/20, 64%) health system executives describing their care team as “tired and frustrated” with the COVID-19 journey. About one-third (6/20, 32%) were in a state of “acceptance of the new normal,” and 5% (1/20) described their status as “return to hope.”

Health system executives commented that, although the future outlook is uncertain, a proactive mindset is needed to create the future they want for their patients and their health care providers. This includes key actions such as executing and building on the established continuum of virtual care; advocating for payment, site flexibility, and reimbursement parity for virtual care; and continued engagement and boldness from leadership to evolve care beyond established models. Such care models are evolving toward a hybrid model of virtual, in-person care supported by changing the mindset of both patients and health care providers of what the new patient-health care provider relationship is seen as across the continuum of the patient care journey and not just episodic care as it may have been before the pandemic.

## Discussion

### Principal Findings

Through surveys and interviews with executives from many of the largest health systems in the United States, this study assessed and makes recommendations on the strategy, business model, implementation, and future considerations for implementing virtual health solutions in the United States. The key findings were (1) the main impacts of increased demand for virtual health solutions on health systems were increased access and flexibility and lower costs of care delivery; (2) health system executives commonly believe that virtual health is best supported through value-based payment models; and (3) almost all health systems expect to continue growing their virtual health offerings for the foreseeable future, with reimbursement being the key challenge to virtual health scaling.

A robust, patient-centric virtual health offering is now a standard expectation for health system executives. Health systems are evaluating whether their current virtual health system meets the differing needs of the system, health care providers, and patients and are identifying the mission-critical goals of a long-term virtual health strategy. Although the external shock of COVID-19 disrupted care models, all participating health systems are expecting to continue virtual care moving forward.

A key consideration in maintaining virtual care is to establish its safety and efficacy relative to traditional in-person care. The efficacy and safety of virtual care would be dependent on the nature of the solution and circumstances of use. For example, a Cochrane review of admission avoidance–hospital-at-home programs showed no differences in mortality between patients treated using admission avoidance–hospital-at-home programs compared with conventional inpatient hospital care [19]. On the other hand, virtual solutions such as a chatbot have proved more controversial, and experts have raised concerns about efficacy [20]. Delivering care virtually can also impact the patient-health care provider relationship, as physical touch and eye contact can communicate interest and empathy. In one study, patients who did not previously have a relationship with their health care provider reported feeling less comfortable with clinical video telehealth than those who had previously visited in person [21].

Current virtual health platforms were sufficient for participating health systems’ COVID-19 response but far from perfect. These health systems are reevaluating their current platforms, processes, and strategy to develop a long-term approach to virtual health that addresses key challenges, including adjusting clinical and operational workflows that meet clinical and operational needs with one user-friendly, integrated, scalable platform to ensure patients’ access to technology and avoid exacerbating existing health equity disparities. Early adopters of new medical technologies commonly have high socioeconomic status due to differences in access (the digital divide) and differences in use (due to factors such as health literacy) [22]. The transition towards increased adoption of virtual health solutions has the potential to either relieve or exacerbate existing health care disparities. The decisions that health systems make now in the types of solutions they offer, and the strategies they choose to minimize disparities, are important in shaping health care equity for the future. For example, apps that are difficult to use could make accessing care disproportionately more difficult for people who are older, have a lower level of education, or are from a diverse racial or ethnic background, who may have lower digital literacy [23]. Alternatively, health equity disparities may decrease over time for technologies that simplify treatment, as innovations gradually diffuse through society [22]. Another concern is fragmentation of patient care due to the expansion of non-health system–affiliated virtual care options. This could erode relationships between health care providers and patients and reduce the effectiveness of care due to a lack of communication between affiliated and nonaffiliated health care providers. There is also a lack of clarity on which clinical and financial metrics are most valuable to validate virtual health’s cost and efficacy compared with in-person care. With one estimate showing that US physicians spend approximately 15 hours per week on quality metrics [24], it is important to focus on the most valuable metrics to minimize the time and effort these require.

Participating health systems report significant uncertainty around changes to payment models and regulatory policy (eg, reduction in payment parity, eliminating payment for phone-only visits, persistence of site-of-care, and state licensing restrictions) as they try to plan for the financial sustainability of their virtual health offerings. A lack of payment parity for virtual and in-person visits could be a disincentive to offer virtual visits, because if insurers reimburse telehealth appointments at a lower rate than in-person visits, health systems may have to operate telehealth services at a loss or not offer these services at all. Regulatory policies also differ substantially by state; for example, 43 states and Washington DC have payment coverage laws for telehealth, but only a subset of these have payment parity laws [25]. Health system executives have a role to play in shaping the financial sustainability of virtual health solutions going forward, as they can proactively advocate for continuation of many of the regulations and payment structures that have enabled increased virtual health adoption during the pandemic.

Health system executives are concerned that current virtual health modalities are not sustainable if regulations regress toward prepandemic status; however, many note that accelerated movement toward value-based payment models would support virtual health expansion. Health systems that proactively seek alternate payment models could benefit in the long-term with payment structures that are more sustainable in a post-COVID-19 world. The use of value-based payment models is predicted to increase due to incentives created by the 2015 Medicare Access and CHIP Reauthorization Act [26]. Under this act, stakeholders are able to propose alternative payment models to the Physician-Focused Payment Model Technical Advisory Committee. An example of an alternative payment model is the bundled hospital-at-home and 30-day postacute transitional care program at the Icahn School of Medicine at Mount Sinai in New York City, which led to improved patient outcomes and care ratings compared with traditional inpatient care [27]. In this payment model, health care providers were able to charge a base payment for hospital-at-home services and fee-for-service charges for other services, demonstrating the feasibility of this alternate payment model.

To establish a sustainable, enterprise-wide approach to virtual health that addresses the challenges and barriers laid out in this research, health systems are working through key components, including governance structures, finance, data/IT, and clinical operations. As health systems evolve in their virtual health maturity, governance structures tend to become more centralized, with the most mature health systems having explicit C-level support for virtual health. Virtual health budgets become more defined and high-level, wherein advanced and innovative health systems have system-level budget lines for virtual health, and work with payers to develop and advocate for virtual health payment structures. With increasing maturity, data and IT systems become more advanced, and health systems proactively seek cutting-edge technologies, move beyond standard metrics, develop interconnectivity between platforms, and proactively monitor for cybersecurity threats. Additionally, health care providers become increasingly empowered and able to seamlessly integrate virtual and in-person care. As health systems evolve in their virtual health maturity, their market impact is expected to increase.

### Strengths and Limitations

This analysis has several strengths and limitations. One strength is the breadth of executive types that inform this study. Participants included executives from the 3 categories of clinical, operations, and data/IT, which may assist in understanding virtual health from different perspectives. In addition, survey participants represent a variety of health systems, differing by size, region, degree of government pay, and virtual health maturity level. This even distribution of health system features suggests that the sample is likely to be representative of the health system landscape in the United States, which is further supported by the substantial portion of the total US health system market represented by these systems. These surveys do, however, only represent a subset of systems, and these findings may not be applicable to every health system. Also, these findings represent the views of individual executives, and there may be differences of opinion within individual health systems. In addition, by including only US-based health systems, some of the issues raised are specific to the United States, such as concerns around state licensing requirements. Although this analysis is limited to US health systems, the COVID-19 pandemic has precipitated a trend toward increased adoption of virtual health in countries around the world [11], and it is likely that many of the trends and challenges highlighted by this study would be applicable in other countries. Additionally, the timing of the study in late 2020 allows the authors to only document health systems’ early response to the pandemic.

### Conclusions

The COVID-19 pandemic saw a substantial increase in virtual health adoption, and this increase is expected to continue postpandemic. Consequently, health systems are reevaluating their current platforms, processes, and strategy to develop a sustainable, long-term approach to virtual health. Health system leaders need a proactive mindset to create the future they want for their patients and their health care providers, including executing and building on the established continuum of virtual care; advocating for payment, site flexibility, and reimbursement parity for virtual care; and continued engagement and boldness to evolve care beyond established models.

## Appendix

Multimedia Appendix 1 Quantitative survey instrument.

Multimedia Appendix 2 Qualitative interview guide.

## Abbreviations

CMO: chief medical officer
EMR: electronic medical record
IT: information technology
mHealth: mobile health
TOR: total operating revenue
