# Supplementary material for: Accelerating Virtual Health Implementation Following the COVID-19 Pandemic: Questionnaire Study
Source: JMIR Form Res. 2022 May 16;6(5):e32819. doi: 10.2196/32819 (PMC9116483; doi:10.2196/32819)
Supplement: Multimedia Appendix 1 [file formative_v6i5e32819_app1.docx]

For the purposes of this survey, The Academy is defining virtual health to include:

- **Live (synchronous) videoconferencing:** a two-way audiovisual link between a patient and a care provider
- **Store-and-forward (asynchronous) care delivery:** transmission of a recorded health history to a health practitioner or patient
- **Remote patient monitoring (RPM):** the use of connected electronic tools to record personal health and medical data in one location for review by a provider in another location, usually at a different time.
- **Mobile health (mHealth**): health care and public health information provided through mobile devices. The information may include general educational information, targeted texts, and notifications about disease outbreaks.

# General Quantitative Survey

*Target Audience: all participating executive cohorts*

1. How would you describe your health system’s virtual health governance structure?
   1. No clear governance structure
   2. Defined but decentralized governance structure
   3. Defined and centralized governance structure
   4. Other: ____________________
2. Pre-COVID, where did your organization fall on the spectrum in pursuing the following virtual health modalities?
   1. Synchronous care (e.g., videoconferencing)
   2. Asynchronous care (e.g., store and forward videoconferencing, eVisit)
   3. Remote patient monitoring
   4. Mobile health
      1. Ranking: 1 (Conservative) to 5 (Innovator/early adopter)
3. Beyond the immediate response to the pandemic, to what extent do you plan to scale your virtual health services across the next 6 -12 months?
   1. We **plan to continue to grow** most or all of our virtual health offerings for the foreseeable future.
   2. We **plan to** **maintain** most or all of our virtual health offerings at close to their current state.
   3. We **plan to** **roll back** some of our virtual health offerings as some care shifts back towards in-person.
   4. Other: ___________
4. How would you describe your health system’s virtual health budget?
   1. No defined virtual health budget
   2. Disparate budgets sit across various functions/levels
   3. Defined virtual health budgets live within broader service line budgets
   4. Defined budget for virtual health at the system level
   5. Other: ___________
5. How would you rate your health system’s proficiency in the following categories in relation to virtual health?
   1. Virtual health technology
   2. Data analytics capabilities
   3. Cybersecurity
   4. Interoperability
      1. 1 In early stages
      2. 2 Functional but not at goal
      3. 3 At goal and advancing
      4. 4 Innovating at the cutting edge
6. How do you anticipate adjusting your approach to virtual health moving forward?
   1. Our existing virtual health system is **sufficient for most or all of our needs**, and we are focused on continuing to upgrade and optimize it going forward.
   2. Our existing virtual health system **meets some of our needs**, but we are open to/seeking other systems which may offer a better overall fit.
   3. Our existing virtual health systems is **not sufficient for our needs going forward**, and we will need to heavily modify it or switch to a different system.
   4. Other: _____________
7. What software(s) is your health system using for virtual health? Check all that apply.
   1. Zoom
   2. EMR provider
   3. Skype
   4. Webex
   5. Teledoc
   6. Vidyo
   7. Amwell
   8. Intouch
   9. Homegrown solution
   10. Other: ______________
8. How would you rate your health system’s success with the following?
   1. Virtual health adoption among your physicians
   2. Integration of virtual health into existing clinical workflows
      1. 5 Very successful
      2. 4
      3. 3
      4. 2
      5. 1 Not at all successful
9. To what extent do each of the following program elements present a challenge to scaling your virtual health enterprise? (Rank: 1 – Not a challenge, 5 – Significant challenge)
   1. Virtual health technology platform(s)
   2. EHR integration
   3. Data security
   4. Complete documentation and data capture
   5. Clinical workflows
   6. Cost to support service
   7. Defining roles for in-person and virtual care delivery
   8. Physician resistance
   9. Patient disinterest
   10. Patient access to technology/literacy
   11. Reimbursement
   12. Other: _______________
10. To what degree does your current virtual health infrastructure meet your needs for your virtual health strategy for your 2+ year vision?
11. 5 Completely meets our needs
12. 4
13. 3
14. 2
15. 1 Does not meet our needs
16. How consumer-friendly would you say your virtual health system is?
17. 5 Very consumer-friendly
18. 4
19. 3
20. 2
21. 1 Not consumer friendly

# Clinical

*Target audience: CMO, Medical Group Leader, CNO*

1. What proportion of your providers’ virtual health interactions are provider-to-provider (consultations between providers, primarily for specialty and subspecialty care)?
   1. 0-10% provider-to-provider
   2. 10-20% provider-to-provider
   3. 20-30% provider-to-provider
   4. 30-40% provider-to-provider
   5. 40-50% provider-to-provider
   6. Above 50% provider-to-provider
2. What percent of your virtual health solutions in use are primarily synchronous versus asynchronous?
   1. Synchronous visits: _______% of virtual health utilization
   2. Asynchronous information-sharing: _____________% of virtual health utilization
3. What percent of patient visits were delivered through virtual health solutions at the peak of your health system’s COVID surge? What percent are currently delivered through virtual health?
   1. Peak utilization: ______________
   2. Current utilization: ___________
4. Has your health system established a target for proportion of care delivered through telehealth moving forward? If so, what is your target utilization?
   1. 0-10%
   2. 10-20%
   3. 20-30%
   4. 30-50%
   5. 50% or more

# Strategy and Operations

*Target Audience: CFO, COO, CSO*

1. What factors are most important in making a go-forward decision on a virtual health solution? (Rank top 3)
   1. The set-up and activation costs of the system
   2. The ongoing maintenance costs and fees associated with the system
   3. The user-friendliness of the system for providers
   4. The user-friendliness of the system for patients
   5. The ease of billing
   6. The ease of integrating the system with an EMR and sharing information
   7. The ease of integrating with the current virtual health system
   8. Analytic capabilities included with the system
   9. The scalability of the system
   10. Input from providers and staff
   11. Other: ___________________________
2. Has your health system calculated an ROI on your current virtual health solutions?
3. Yes, we have calculated ROI
4. We are in the process of evaluating ROI
5. We have not been able to achieve ROI
6. In light of your virtual health strategy, which of the following cost management tactics have you recently implemented or plan to implement across the next 6-12 months? Check all that apply.
   1. Redesign clinician compensation
   2. Workforce adjustments (e.g., reducing clinic support staff)
   3. Reducing clinic square footage (existing space or modifying future space plans)
   4. Implement technology to automate certain tasks (e.g., clinical documentation during virtual visits)
   5. Other: ____________________
7. How has your health systems revenue cycle team processed virtual engagements?
8. Our revenue cycle function struggles to process virtual health engagements
9. Our revenue cycle function can adequately process virtual health engagements
10. Our revenue cycle function seamlessly integrated virtual health engagements into normal revenue cycle workflow

# IT/Data

*Target audience: CMIO, CNIO, CIO*

1. Which metrics is your health system using to track the success of virtual health services? Check all that apply.
   1. Patient satisfaction
   2. Physician engagement
   3. Visit time
   4. Patient wait time
   5. Percent of clinicians using virtual health solutions
   6. Accuracy of diagnosis
   7. Patient retention
   8. Clinician retention
   9. Treatment plan adherence
   10. Return on investment
   11. Clarity of care instructions
   12. Platform ease of use
   13. Net promoter score
   14. Service uptime
   15. Number of connectivity issues
   16. Other: ______________
2. Are you currently planning on moving your organization to a single platform across the entire system?
   1. We currently use one solution across the organization
   2. We currently use multiple platforms, but expect to move to one solution in the next 1-2 years
   3. We currently use multiple platforms, and are evaluating whether to move to one solution in the future
   4. We currently use multiple platforms and expect to continue with multiple solutions moving forward
3. How successfully is your health system able to integrate data from virtual health solutions into existing EHR systems?
   1. 5 Very successfully – fully integrated
   2. 4
   3. 3
   4. 2
   5. 1 Unsuccessful – not at all integrated
