# Supplementary material for: Accelerating Virtual Health Implementation Following the COVID-19 Pandemic: Questionnaire Study
Source: JMIR Form Res. 2022 May 16;6(5):e32819. doi: 10.2196/32819 (PMC9116483; doi:10.2196/32819)
Supplement: Multimedia Appendix 2 [file formative_v6i5e32819_app2.docx]

# Qualitative Interview Guide

For the purposes of this conversation, The Academy is defining virtual health to include:

- **Live (synchronous) videoconferencing:** a two-way audiovisual link between a patient and a care provider
- **Store-and-forward (asynchronous) care delivery:** transmission of a recorded health history to a health practitioner or patient
- **Remote patient monitoring (RPM):** the use of connected electronic tools to record personal health and medical data in one location for review by a provider in another location, usually at a different time.
- **Mobile health (mHealth**): health care and public health information provided through mobile devices. The information may include general educational information, targeted texts, and notifications about disease outbreaks.

*Note: Interview questions are intended as a guide for discussion, not necessarily an exhaustive list – the conversation may center on specific topics depending on time constraints and topic relevance.*

## Strategy

*Target audience: C-suite executives*

1. What virtual health modalities was your system using widely pre-COVID, and what virtual health solutions have you implemented post-COVID?
   1. Across which specialties and for which services?
   2. Are you using mobile apps as part of your virtual health strategy? If so, how?
   3. What new developments in virtual health has your health system made over the past four months? Were these changes only in response to COVID or for another reason?
   4. Where did you see the greatest growth in utilization?
2. What virtual health platform(s) does your health system currently use, and why?
   1. What are the advantages and disadvantages of your current platform?
   2. Does your health system use different platforms across the organization (e.g., for provider consults vs. direct-to-consumer)?
3. What are the most important factors your health system analyzes when considering a change in virtual health solution?
   1. What are the barriers in your health system to change to a virtual health solution that’s better than your current one – how much better does it have to be, and in what ways, to make the switching costs worth it?
4. What are the key metrics of success and KPIs your organization is assessing to evaluate your current virtual health programs?
   1. Has your health system evaluated the impact on patient outcomes? What are the financial and operational measures your organization is tracking?
   2. What patient-centered metrics are you tracking (e.g., patient engagement, satisfaction)?
5. Has the switch towards virtual health impacted your approach to other broad strategic issues (e.g., consolidation, workforce transformation)?
   1. Why or why not, and if yes, what kinds of changes are you consolidating and why?
6. Given the evolving payment environment, what have been the “no-regrets” investments your health system has made in virtual health?
   1. What are the less certain investments?
7. How is your health system managing regulatory uncertainty around telehealth (e.g., payment parity, interstate licensing) as you develop long-term strategy?
   1. What are the critical policy changes that would be needed to make virtual health sustainable for your health system?
   2. In what ways has the pandemic pushed your organization to take a more active, outward-facing role in advocating for the regulatory actions that would facilitate your use of virtual health?

## Structure

*Target audience: C-suite executives, clinical (e.g., CMO, MG leader)*

1. Who owns decision-making for your organization’s virtual health strategy? What is the governance structure of the decision-making body?
   1. How are decisions cascaded down through the organization?
   2. How is the decision-making process communicated through the organization to facilitate effective gathering of innovative ideas?
   3. Are there separate governance structures for in-person and virtual care delivery?
   4. How are they aligned and coordinated?
   5. How do you expect the governance and leadership structures supporting virtual health to evolve in the future?
2. How does your physician alignment model enable operationalizing of your virtual health strategy?
   1. Does the effect of your physician alignment model differ between physicians who are: employed/affiliated/non-affiliated but with admitting privileges?
   2. How do you plan to balance workforce needs, particularly among specialists?
3. What do new care pathways look like using virtual health tools?
   1. What resources exist for providing guidance on treatment guidelines/care pathways when using virtual health?
   2. Besides physicians, what other care team members leverage your virtual health platforms?
4. What aspects of care delivery do you anticipate will NOT move into the virtual space in the next 2 years?
   1. Which specialties do you anticipate will utilize virtual health most in the next 2 years? Where is the greatest opportunity for expansion?

## Implementation

*Target audience: Telehealth/virtual health leads (e.g., Director of Telehealth, Virtual Care Operations Director)*

1. Please describe the implementation process for integrating new virtual health solutions into your health system. How did this process evolve/change with the onset of COVID-19?
   1. How did your organization scale virtual health solutions across the organization?
   2. Who are the key decision-makers for implementation of virtual health?
   3. Who owns each piece of the implementation of your virtual health strategy?
2. For the following questions, please **provide examples or process specifics that your system uses to ensure efficient and effective implementation of virtual health**:
3. Do you follow any specific implementation framework or process routinely within your health system (e.g., Lean, Agile, Waterfall, etc.)?

1. **Opportunity/Problem or Issue finding**: Describe how you find the right opportunities to redesign care utilizing virtual health and technology.
   - 1. How do you identify and define the opportunity and potential intervention?
     2. What data sources and issue-finding tools or templates do you utilize?
2. **Insight finding**: After identifying an opportunity where virtual health solutions could be effective.
   - 1. How do you gather and synthesize additional insights on the opportunity to ensure the intervention you design will be impactful?
     2. Do you utilize specific data sources, surveys, focus groups to help with this?
3. **Solution finding**:
   - 1. What do you do to ensure the intervention(s) you chose to implement are the most efficient/effective solutions to effectively redesign care and close care gaps while utilizing virtual health?
     2. Do you follow any processes or standard activities to define or refine your solution/intervention (e.g. 5 whys, Fishbone/Cause and Effect, etc.) prior to solution implementation?
4. **Solution Implementation**:
   - 1. How do you create a new clinical workflow for a new care process or intervention?
     2. Who does this?
     3. How are you changing/optimizing workflows to support virtual health?
     4. When comparing new workflows (post intervention) to old (pre-intervention) how do you prioritize technology solutions based on the impact and difficulty of implementation?
     5. How do you ensure care processes are efficient and effective for care teams and within the EHR?
     6. To what extent are new workflows integrated into the EHR? To what extent is there clinician continuity for patients across virtual/in-person care settings?
5. **Improving and Optimizing**:
   - 1. After implementing a new clinical workflow that includes virtual health how do you iterate and test the new care process to ensure scaling throughout your health system is efficient and effective?
     2. Can you provide a few examples on how you measure results?
6. What have been the greatest challenges in implementing and scaling a virtual health program? What have been your biggest “wins”?
7. Did your health system develop any best practices for accelerating the implementation and scaling process for virtual health solutions?
8. What tools, processes, or solutions were particularly effective at helping your health system grow your program at an enterprise level?
9. How has your health system acquired stakeholder buy-in for the implementation of virtual health programs?
10. What were the clinician, patient, and operational education requirements?
11. How did education and buy-in vary across different solutions or virtual health use cases?
12. Has the shift towards virtual health changed your approach to employee professional development and engagement?
    1. How has it changed your concept of your workforce and your internal culture? Are these changes you’re hoping to maintain in the future?

## Finance

*Target audience: C-suite, finance and operations (e.g., CFO, COO, VP Finance)*

1. How aggressive are you being in re-shaping your budget for end of this year, 2021, and beyond, to incorporate the changes brought by the expansion of virtual health?
2. Have you defined a budget dedicated to implementing, scaling, and optimizing virtual health solutions over the next year?
3. How aggressive are you being on transforming staffing and compensation models to align with your vision/strategy for virtual health? What are the primary changes you are considering?
4. Is the current rate of virtual health visits financially sustainable for your health system?
5. Assuming that the current proportion of virtual visits holds, along with current reimbursement rates, how sustainable is that arrangement for your health system?

20. Does your organization measure ROI for virtual health solutions? Which metrics are the most

important?

- 1. If you have been able to calculate an ROI, is that ROI positive? How does it compare to in-person services that virtual health has replaced?

1. Are your virtual health modalities primarily focused on replacing revenue that has been lost due to the pandemic, or on providing new channels of revenue on services you were not previously delivering or patients you had not previously reached?
   1. For replacement revenue, are you currently achieving a 1:1 replacement (i.e. a telehealth visit brings in the same revenue as an in-person visit) across the various services and/or populations?
   2. How do you anticipate your health systems’ virtual health strategy will impact your market share?
2. How much of your future approach towards virtual health will be building versus buying?
   1. Which aspects of your virtual health systems do you anticipate contracting out versus keeping in-house?
3. To what extent has virtual health reimbursement parity impacted your health system’s decision to advance virtual health initiatives?
   1. To what extent would the reversal of reimbursement parity and the implementation of telehealth waivers impact your health system’s approach to virtual health expansion moving forward?

## Data

*Target Audience: C-suite executives, IT (e.g., CMIO, CNIO)*

1. How is your health system ensuring that you:

- Have sufficiently robust, detailed, and integrated data-collection systems to collect the information you need to reduce care disparities and unwanted clinical variation
- Protect patients’ data privacy and ensure patients’ confidence in your security measures

1. How have you restructured your data infrastructure to support virtual health? What are the key metrics and data points you are collecting and evaluation from your virtual health solutions?
   1. To what extent is virtual health data aggregated into dashboards at a system-level?
   2. How are you structuring your virtual health data to comply with new data-sharing regulations? Are you approaching virtual health data differently from other data sources at your health system?
2. Are you integrating any patient-generated health data into your data collection mechanisms?
3. What challenges and concerns (e.g. legal concerns around requirements to use the data once collected, or concerns around clinical utility/reliability) do you have around collecting and using these data?

1. As you look towards the future, what role will Artificial Intelligence and Machine Learning tools play in your virtual health approach?
2. As a Health System how are you preparing for the 21^st^ Century Cures Act’s interoperability and information blocking rule? How will this change the dynamic of information sharing with 3^rd^ party virtual health tools you use?

## Consumerism

*Target Audience: C-suite executives, strategy and operations (e.g., CSO, COO)*

1. How do you view new non-health system entrants to the virtual health space (Apple, Google, Amazon, Haven, Zoom) - as competitors, potential partners, or both? What are you learning from them to inform making your virtual health strategy consumer-friendly/consumer focused?
2. How are you ensuring equitable access to virtual health across various patient populations? Are you targeting offerings to particular patient cohorts based on established need? How are you supporting your patients who are having difficulty navigating the technology?
3. Are you conducting consumer research to evaluate the patient needs/preferences on virtual health, and the success of virtual health implementation and your current offerings?
   1. What have you learned from patient feedback about what works well and what needs to be improved in your virtual health system?
4. How do you see recent (e.g., 21st Century Cures Act) and future interoperability and data sharing legislation impacting patient’s care delivery journey within the context of your health system?
